# Bystander Intervention in Football and Sports. A Quasi-Experimental Feasibility Study of a Bystander Violence Prevention Program in the United Kingdom

**DOI:** 10.1177/08862605241239452

**Published:** 2024-03-26

**Authors:** Anastasiia G. Kovalenko, Rachel A. Fenton

**Affiliations:** 1 University of Exeter, Devon, UK

**Keywords:** domestic violence, sexual assault, prevention, intervention, sexual harassment

## Abstract

In recent years, social campaigns and high-profile cases have brought increased attention to violence against women. Athletes can be role models, shaping both prosocial and antisocial attitudes. Their engagement in violence prevention could be an effective tool to tackle violence against women through bystander intervention. This part of a mixed-method feasibility study reports on the quantitative evaluation of an evidence-led bystander program, Football Onside, implemented at a football club in South West England in June 2018 to February 2020. The study employed a quasi-experimental design with intervention and control groups. Football coaches and club members (*n* = 50) completed measures of rape and domestic abuse myths, bystander intent and efficacy, self-reported bystander behaviors, readiness for change, perceptions of peer helping and myth acceptance, law knowledge, and program evaluation. Fidelity was also assessed. The analysis compared between- and within-group differences in mean changes over time using mixed-effects models. Participant ratings of learning outcomes were high, and fidelity was maintained throughout the intervention. Between-group comparison revealed mixed results, with greater improvements in the intervention group for bystander intent and efficacy at post-test and follow-up, domestic abuse myths at post-test, and rape myth acceptance at follow-up. Model contrasts for within intervention group revealed improvements in rape and domestic abuse myth acceptance, bystander intent and efficacy, perceived law knowledge at both time points, and perceived peer myths and helping at post-test. At follow-up, intervention participants reported significantly higher engagement in bystander behaviors. No significant effects were found for perceived importance of legal knowledge. Our research highlights the potential efficacy of a bystander program tailored for football club members. Cluster-randomized control trials are now required to examine bystander attitudes and behavior change processes among professional athletes.

The United Kingdom has entered a pivotal moment in history in relation to combatting violence against women and girls (VAWG) in the wake of one particular high-profile abduction and murder in March 2021, which has invigorated the national conversation and consciousness. Understood as a cause and consequence of gender inequality, VAWG is a public health epidemic and a global human rights issue (UN, 1993; WHO, 2010). In the United Kingdom, for example, one in four women experience domestic abuse, and one in five experience any kind of sexual assault in their lifetime (Home Office, 2019). Since the beginning of the COVID-19 pandemic, VAWG, especially domestic abuse, has intensified globally (UN Women, 2021) and has been referred to as “the shadow pandemic.” For example, in April to June 2020, roughly one-fifth (21%, 20%, and 19%) of all offences recorded by the police in the United Kingdom were flagged as domestic abuse, which is a 5%-point increase compared with the same period in the previous year (ONS, 2020). In the year ending March 2022, the police recorded the highest number of sexual offences in England and Wales, with 86% sexual assault victims being female (ONS, 2023a), and it is estimated that only one in five cases is reported to the police (ONS, 2020). Similarly, in the year ending March 2023, 73.5% of police-recorded domestic abuse victims in England and Wales were female (ONS, 2023b). The elimination of VAWG is a strategic priority both nationally (VAWG Strategy—Home Office) and in terms of the United Kingdom’s international legal obligations (UN, Convention on the Elimination of all Forms of Discrimination Against Women (CEDAW), Council of Europe). Yet while some positive legislative initiatives are being delivered, such as the Domestic Abuse Act 2021, the criminal justice system reveals an ever-increasing inability to provide redress with charging, prosecution, and convictions for sexual offences at a 10-year low (CPS, 2019). The focus on prevention and, in particular, how to engage men in prevention has thus never been more relevant in UK society than in this potentially transformative moment.

One prevention strategy gaining traction in the United Kingdom is bystander intervention. Bystanders can play an important role in the prevention of VAWG as agents of cultural change who intervene to disrupt violence and the social norms that facilitate it (Banyard et al., 2004; Orchowski & Berkowitz, 2022). The historical cultural prominence of sport—and, in the United Kingdom, of football (soccer) in particular—and its vindication of traditional models of masculinity linked to violence (Adams, 2011) render it an “unparalleled platform” (Katz, 2018) for VAWG prevention. There is a paucity of knowledge about bystander approaches and sports settings, with no studies to date evaluating the bystander approach in a professional sport setting in the United Kingdom and only very limited evidence elsewhere. With this gap in mind, this study seeks to add to the literature by presenting findings from a controlled quasi-experimental feasibility study of a bystander program, “Football Onside,” with follow-up at 9 months. “Football Onside” is a bystander intervention for the prevention of sexual and domestic abuse, tailored to a male-dominated professional football setting in the United Kingdom for the first time.

## Sports, Masculinity, and VAWG

There is a complex, intersecting, and longstanding association between sport, misogynistic or hegemonic masculinity, and the reproduction of violence-supportive norms and violence perpetration (Dyson & Flood, 2008; Flood, 2011). A body of literature has found that male college athletes have a higher affinity for rape myth acceptance (Boeringer, 1999; Bogen et al., 2020) and are an at-risk group for sexual violence (SV) perpetration (Crosset et al., 1996; McCray, 2015; Young et al., 2017) and victimization (Cheever & Eisenberg, 2022).

Male professional sports settings have been the sites for high-profile incidents (see Katz, 2018), and some surveys have shown an increasing number of reports of rape and domestic abuse cases among professional athletes in contact sports (NBC, 2014; O’Hear, 2001). In the United Kingdom, trials of professional sportsmen for rape have fueled high-profile public debate and controversy within the legal community (McGlynn, 2018). In addition to prominent incidents, very limited research has indicated that domestic abuse rates may increase after high stakes sporting events such as the World Cup and Euro Cup (Kirby et al., 2013; Trendl et al., 2021) and national rival team football matches (Williams et al., 2013). This has, however, been contentious, in particular, due to the heterogeneity of studies, the challenges in measuring, recording, and reporting national domestic violence (DV) data, the lack of evidence outside the United Kingdom and North America, and the limited assessment of the contributing risk factors (Forsdike et al., 2022).

Educators are agreed about the importance of engaging men in prevention efforts (Flood, 2011). As sports define the cultural mainstream with team sports allowing men “to do [] gender in the most culturally esteemed way” (Adams, 2011, p. 580), sports settings simultaneously occupy a critical space and present an opportunity for prevention work. Athletes can be positive societal role models (O’Donohue & Schewe, 2019), promoting prosocial behaviors and healthy ways to express masculinity and disapproval of the social norms, which shore up VAWG (Carlson et al., 2015; Katz et al., 2011).

Coaches can be influential positive nonparental role models (Fraser-Thomas et al., 2008). Further, sports teams may be encoded with strong social bonds, which can be mobilized to promote positive intervention (McMahon & Farmer, 2009). Indeed, social expectations around masculinity are related to both perpetration and men’s willingness and likelihood to intervene as bystanders (Brown & Messman-Moore, 2010; Carlson, 2008; Fabiano et al., 2003; Schwartz & DeKeseredy, 2008). Consequently, there is a well-established history of working with athletes and coaches as “exemplars of traditional masculine success” (Katz, 2018) to prevent VAWG (e.g., Mentors in Violence Prevention (MVP)—Katz, 1995, 2018; Coaching Boys into Men (CBIM)—Miller et al., 2012; Wingman 101—Exner-Cortens & Cummings, 2017).

## Bystander Interventions

Sports settings align well with bystander intervention because the bystander approach focuses on prosocial framing and engaging men as allies (Flood, 2011). The increasingly promising evidence base is situated predominantly in school and college settings in the United States (Kettrey & Marx, 2019; Kovalenko et al., 2022; Mujal et al., 2021). In the United Kingdom, the evidence base is in its infancy, but there is some preliminary evidence in school and university settings (Bovill & White, 2022; Fenton & Mott, 2018; Williams & Neville, 2017) and, more recently, in a general population setting (Gainsbury et al., 2020). Overall, the literature reports improvements across a range of associated measures (for reviews see Kettrey & Marx, 2019; Kovalenko et al., 2022; Mujal et al., 2021). However, there are limitations: only a small number of evaluations include a comparison group, backlash is rarely measured, and the majority have a follow-up period shorter than 7 months, which might explain why few report higher levels of self-reported bystander behaviors (Kovalenko et al., 2022; Mujal et al., 2021). Bystander programs targeting athletes have been predominantly implemented in student populations and report improved bystander intent and efficacy, rape myth acceptance, and perpetration rates (Garrity, 2011; Jaime et al., 2018; Miller et al., 2012; Moynihan et al., 2011b). The findings for behavior change have been mixed, showing small improvements or no significant change (Exner-Cortens & Cummings, 2017; Garrity, 2011). Research evidence on bystander intervention in professional sports, however, is extremely limited (Corboz et al., 2016; Powell, 2011) and lacks rigorous evaluation.

Bystander interventions often intend to increase the knowledge of substantive law, but this is undertheorized in terms of its contribution to change and is rarely reported on (Fenton et al., 2016). Backlash, understood as movement by more than one standard deviation (*SD*) in the undesired direction (Moynihan et al., 2011a), is also rarely reported but is important, as interventions may produce unintended effects.

## Community Trusts

Capitalizing on the normative status of football in society and the extent of outreach by football clubs in the United Kingdom (via Community Trusts^1^), the CEO of a Community Trust was approached as a potential partner in the development, implementation and evaluation of the “Football Onside” program.

Researchers may encounter institutional and cultural resistance implementing violence prevention programs (Brackenridge, 2002; Parent & Fortier, 2018). Nevertheless, overcoming these barriers is possible with strong support from team leaders, their active engagement in the development and implementation of initiatives, and collaborations with external organizations and communities (Parent & Fortier, 2018). It is therefore not unexpected that the enthusiasm, motivation, and commitment of the CEO were instrumental in the realization of the project, and steering group comprising victim organizations, public health, local council, and other professionals in the area was actively engaged throughout the project. The intervention and the study was designed as a response to both need and opportunity for a bystander VAWG prevention program specifically targeting professional players, coaches and staff outreaching into the wider community through football.

## Football Onside

Football Onside is a feminist, gender-transformative, and social-justice-informed bystander leadership intervention. The program is theoretically and pedagogically underpinned by previous research, documented elsewhere (Fenton & Mott, 2017; 2018; Fenton et al., forthcoming). The theoretical framework is Latané and Darley’s (1970) situational model, which explains the bystander decision-making process from noticing an event and identifying it as problematic, to taking responsibility, feeling confident, and possessing the necessary skills to act, through to subsequent intervention. Prochaska and DiClemente’s (1986) model of behavior change was utilized (Fenton & Mott, 2018), and Nation et al.’s (2003) principles for effective prevention programs, for both content and delivery including varied teaching methods, dosage, being theory-driven and socioculturally relevant to participants, and delivered by well-trained staff, were followed. In order to ensure that the content was of maximal salience to participants (Nation et al., 2003), a focus group with the Community Trust staff was held to inform content development. Content included specific requests for information on law, including materials on grooming and child sex offences, which was implemented both in the content and in an additional take-home booklet. Further perusal of the baseline data informed program development, for example, concentrating on the rape myths participants most believed in and providing feedback on participants own norm misperceptions. Content was designed to explore performative masculinities and sport, prevalence, impact and myths surrounding SV and DV, consent, and bystander skills-building (Table S1, Supplemental Appendix).

Trainers were recruited from, or recommended by, local victims’ organizations. Facilitator training was conducted face to face with the two academics who designed the program, and all materials including detailed facilitator notes were provided in advance. The training consisted of going through the slides and notes over the course of a half day. It is worth noting that there was some resistance to the idea that trainers for victims’ organizations could need any further training.

This study sought to explore the effects of Football Onside in a sports setting outside a student population for the first time in the United Kingdom. To address some of the limitations in the evaluation literature, we adopted a nonrandomized design with intervention and control group, with measures at baseline, post-test, and 9-month follow-up. Our primary research aims were to evaluate the feasibility of the Football Onside program and to examine the effect of the program between the intervention and control group. We aimed to explore and report the effects within the intervention group in order to map onto extant evaluations, which did not have a comparison. Our secondary research aim was to examine backlash arising from the intervention.

## Method

### Participants

This study was conducted with two professional Football Club Community Trusts (FCCTs) in South West England from June 2018 to February 2020. Trust 1 received the intervention and Trust 2 acted as control. Of 60 participants initially recruited, nine dropped out after completing the pre-intervention questionnaires due to reasons unrelated to the nature of the study, and one participant, affected by the topic, disclosed during program delivery and was assisted to access professional support. These participants (*n* = 4) in the intervention group and (*n* = 6) in the control group were excluded from the data analysis. The final sample (*n* = 50) consisted of FCCT coaches and managers, football academy students, members of a national personal and social development program, and the regional Football Association.

### Procedure

Members of both Trusts were recruited through emails sent by their senior management. Initially, 12 participants from Trust 1 completed the baseline survey prior to participating in a focus group with the program developers. Measures were taken at baseline, post-test at 1-month post-intervention, and follow-up at 9-months post-intervention. Participants provided written informed consent. Football Onside was implemented in Trust 1 across two groups, each of which received three 2-hour sessions, one week apart in February to March 2019, which were delivered by two trained male and female facilitators together. Fidelity of program delivery was observed. The post-test questionnaire at 1-month contained course evaluation feedback questions. Participants received a £15 voucher at post-test, and an additional £10 voucher at follow-up. Anonymity of participants was maintained using generated ID codes enabling baseline and post-questionnaires to be linked. Trust 2 were offered free delivery of the Football Onside program after final data collection. Ethical approval was granted by the University of Exeter College of Social Sciences and International Studies Ethics Committee.

### Measures

#### Self-Reported Learning Outcomes and Fidelity

Intervention group participants rated the extent to which the program met its learning objectives (improved knowledge, attitudes, likelihood to intervene and confidence) on a Likert-type scale from 1 (“Definitely no”) to 5 (“Definitely yes”). Mean scores for each question were calculated. Fidelity was observed in terms of adherence to the curriculum, quality of delivery, and program attendance. The same observer conducted all observations, completing six fidelity observation logs.

#### The Questionnaire

The survey included validated scales measuring attitudes, intentions, and bystander behaviors around sexual and domestic abuse, designed to map onto the extant literature, as well as questions to gather demographic information. Where necessary language was modified to be relevant to a UK adult audience: “girl” was replaced with “woman,” “campus”/“university” with “my organization” and “groups I work with,” and “community resource” with “professional agency.” Phrases “sexual abuse and intimate partner violence abuse” were replaced with “sexual violence and domestic abuse,” and “911” with “999.”

##### Demographics

Participants were asked their gender, sexual orientation, age, ethnicity, relationship status, if they knew someone affected by sexual or domestic abuse, and prior participation in a prevention program.

##### Rape Myth Acceptance

The Illinois Rape Myth Acceptance Short Form (IRMA) scale was used (McMahon & Farmer, 2011). The participants were asked to rate their beliefs about 19 statements such as “Rape happens when a man’s sex drive gets out of control” on a five-item Likert-type scale with higher scores reflecting greater adherence to rape myths. The mean was calculated, and the scale had a good internal consistency (α = .85).

##### Domestic Violence Myth Acceptance

The Domestic Violence Myth Acceptance scale (DVMAS) was used (Peters, 2008). Participants rated their beliefs in 16 domestic abuse myths, for example, “A lot of domestic violence occurs because women keep on arguing about things with their partners,” on a seven-item Likert-type scale where higher mean score indicated greater acceptance of domestic abuse myths. Internal consistency of the scale was α = .7.

##### Readiness for Change

A subset of items from the Readiness for Change scale (Responsibility and Denial subscales) was used (Banyard et al., 2010, 2014). Where questions asked about one type of violence only, it was adapted to state “domestic abuse or sexual harassment/violence” due to the nature of the study. Item “I think I can do something about sexual violence” was adapted to “I think I can do something about domestic abuse and/or sexual harassment/violence and so I am planning to find out more about what I can do.” The participants answered nine items on a seven-point Likert-type scale, with several items being reverse coded. After reverse coding, the items were averaged so that higher number represented higher readiness for change. Internal consistency was acceptable (α = .6).

##### Bystander Intent

The Intent to Help Friends scale was used (Banyard et al., 2014), along with items from the Bystander Attitude Scale–Revised (BAS-R) (McMahon et al., 2014). Participants rated their likelihood to help in 16 situations, such as “Ring a professional agency to get advice on how to safely challenge a friend, relative, or colleague who is a perpetrator,” on a five-item Likert-type scale where higher mean score indicated higher intent to help. The scale had a high internal consistency (α = .85). One additional item was added to explore participants’ intent to “Look up laws on domestic abuse or sexual harassment/violence,” due to program content and the scale maintained internal consistency at α = .85 level.

##### Bystander Efficacy

The Bystander Efficacy Scale (Banyard et al., 2007) was used to assess confidence to intervene. The scale (*n* = 17) had high internal consistency, α = .89. Participants were asked to rate their level of confidence to perform certain behaviors, for example, “Confidence to speak up to someone who is making excuses for using physical force in a relationship,” on a scale from 0 to 100. A mean score for each participant was calculated, where a higher number represented greater bystander confidence.

##### Reported Behavior

To assess actual behavior change the Bystander Behavior Scale (BBS-R) was used (Banyard et al., 2007; McMahon et al., 2011). The participants reported any bystander behavior performed on a 3-item scale “Yes” (coded as “1”), “Wasn’t in the situation” (“0”), and “No” (“−1”). The scores were summed, with higher scores indicating higher engagement, and the scale (*n* = 16) had high internal consistency (α = .87). Item “Signaled disapproval at sexist comments or jokes by using body language” was added due to program content and “Verbally challenged sexist comments and jokes” was paraphrased from “Challenge a friend who made a sexist joke.” One item was added to assess the law context: “Looked up the law on domestic abuse or sexual harassment/violence.” The scale maintained a high internal consistency α = .87 with the added items. Following McMahon et al. (2017), we assessed the frequency of opportunity (i.e. how many times participants had the opportunity to intervene) and the frequency of bystander intervention (i.e. how many times participants intervened) separately, descriptively analyzing replies “yes” and “no.”

##### Perceptions of Peer Helping

A subset of items from Perceptions of Peer Helping scale (Banyard et al., 2014) and BAS-R (McMahon et al., 2014) was used. We replaced “friends” with “friends, relatives and colleagues of the same gender,” consistent with Gainsbury et al. (2020). Participants rated the likelihood of their peers to perform five certain helping behaviors on a 5-point Likert-type scale. For example, “Approach someone they knew if they thought s/he was in an abusive relationship to let them know they were there to help.” Means for each item were calculated with higher number indicating a greater belief that peers would help. Internal consistency of the scale was moderate (α = .68).

##### Perceptions of Peer Myth Acceptance

A subset of items from DVMAS and IRMA was used, adding a question on program content ("Sexist banter is okay if it’s only a joke”). Participants indicated the proportion (0–100%) of their friends, family, and colleagues of the same gender they thought would agree with each of the seven statements. Internal consistency was α = .6. Baseline scores on Perceived Peer Helping and Peer Myth Acceptance were subsequently used as a pedagogical tool during the intervention to show participants’ actual and perceived norms.

##### Perceptions of Law Knowledge

In accordance with program content, two bespoke items were created to measure perception of law knowledge related to rape and to domestic abuse on a five-item Likert-type scale, for example, “How would you rank your knowledge about the law relating to sexual violence?” Higher scores indicated greater perceived law knowledge.

##### Backlash

We followed Moynihan et al. (2011a), calculating the change in attitude scores over time by at least one standard deviation in the undesired direction. A categorical variable was computed for each participant to see if their score changed or stayed the same.

### Analysis Plan

Data were analyzed using Stata Statistical Software: Release 16. College Station, TX: StataCorp LLC. The dataset was checked for normality of distribution of residuals, and for heteroscedasticity. Sensitivity analysis explored missing items and outliers. We performed a square root transformation for one skewed variable, Bystander Behavior; however, since it did not alter the results, we present untransformed data. Missing data not exceeding 10% of total questions on the scale or subscale were handled using person mean substitution (Shrive et al., 2006); otherwise, the observation was coded as missing for that subscale.

Participants in both groups were compared using descriptive statistics and bivariate tests. We also compared completers with those lost to the 9-month follow-up. Dichotomous variables were analyzed using Chi-square tests, while continuous variables were compared using *t*-tests. Mixed-effects models were conducted to observe changes over time from baseline to post and baseline to follow-up, both between the intervention and control group and within the intervention group. Mixed-effects models are preferable to repeated measures ANOVAs because the approach is more flexible, allows for the analysis of nested cases and predictors (Tabachnick & Fidell, 2013), and handles missing data by estimating parameters based on available individual information (Gueorguieva & Krystal, 2004). Due to small sample size and to reduce the chance of Type 1 error, we adopted a conservative approach with restricted maximum likelihood and Kenward–Roger approximation (Kenward & Roger, 1997; Luke, 2017). Mixed-effects models included time, group, and the interaction between these variables as fixed effects, and random participant effects to account for between-participant variability. The interaction Time × Group reflected the effects of the intervention. We applied appropriate contrasts to observe within-group changes in the intervention group. Standardized effect size based on model-adjusted mean comparison was calculated (Lipsey & Wilson, 2001) using Cohen’s *d* and then Hedges’ *g* to avoid bias due to small sample (Hedges & Olkin, 1985). By way of general guidance, *g* < .2 indicates small effect, *g* = .5 indicates medium effect, and *g* > .8 indicates large effect (Cohen, 1988). Backlash was calculated using Chi-square tests.

## Results

### Participants

Of the 60 initially recruited, 50 (83%) participants completed baseline and post-intervention surveys, and of those, 37 (74%) participants completed follow-up at 9 months. Attrition rates at follow-up were 32.35% (*n* = 11) in the intervention group, and 12.5% (*n* = 2) in the control group, χ^2^(3) = 50, *p* < .001. The majority of participants self-identified as White British (96%), male (84%), and heterosexual (90%), with age ranging between 18 and 58 years (*M* = 31.44, *SD* = 10.945) (Table S2, Supplemental Appendix). Twenty-one participants (42%) reported knowing someone who had been affected by domestic abuse. Five participants (10%) had participated in a DV or SV program before.

There were no substantial differences between the intervention and control groups in terms of demographic variables and baseline scores. There were no significant differences in baseline scores and demographic variables between those who completed the questionnaires at all time points and those who were lost to follow-up. Similarly, participation in the focus group was not associated with any significant differences.

### Primary Research Aims

#### Perceived Learning Outcomes and Fidelity

Participants consistently reported that the program met its learning objectives with mean scores surpassing 4 on every item (*M* = 4.32, 95%CI [4.11, 4.52]) (Table S3 Supplemental Appendix). Mean observer scores across the six sessions ranged from 3.5 to 5.0 (*M* = 4.67; *SD* = 0.61), with one occasion falling below the 4.0 criterion. It became apparent from observation and participant complaint at the first session with the first group that one facilitator was unable to communicate the materials confidently and knowledgeably in accordance with the program learning objectives. Participant interaction with the facilitators in that session was lower. The trained facilitators were immediately replaced by the female–male program developers (second author) and the subsequent two sessions for group one, and the whole program for group two, was unaffected. Fidelity, engagement and attendance were observed to be subsequently high.

#### Effects of the Intervention

##### Between-Group

Table 1 shows model-estimated mean differences and means for between- and within-group changes. We found significant intervention effects at post-test for Domestic Abuse Myth Acceptance, *F*(5, 98.22) = 3.26, *p* = .009, and at follow-up for Rape Myth Acceptance, *F*(4, 98.22) = 6.53, *p* < .001, with greater improvements in the intervention group, and medium effect sizes on both measures. Significant intervention effects were also found in Bystander Intent, F(5, 98.29 = 3.37, *p* = .008, and Bystander Efficacy, *F*(5, 95.52) = 4.34, *p* = .001, at both time points, with greater improvements in the intervention group, and large effect sizes on these measures. No significant effects were found for other measures.

**Table 1.** Mixed-Effects Model Results.

Measure	Intervention groupTrust 1	Control group Trust 2	Between-groupdifference^a^	Within-groupdifference^a^	Effect size^c^
	*M* [95% CI]	*M* [95% CI]	Time × Group I-C [95% CI]	T1-T0, T2-T0 [95% CI]^b^	*d*(*g*)
IRMA					
Baseline	1.87 [1.72, 2.05]	1.83 [1.6, 2.05]			
1 month	1.57 [1.41, 1.72]	1.65 [1.42, 1.88]	−0.12 [−0.37, 0.12]	−0.3 [−0.44, −0.17]**	
9 months	1.49 [1.32, 1.66]	1.75 [1.52, 1.98]	−0.3 [−0.57, −0.04]*	−0.38 [−0.54, −0.22]**	−0.54(−0.53)
DVMAS					
Baseline	2.74 [2.51, 2.97]	2.57 [2.24, 2.9]			
1 month	2.39 [2.16, 2.61]	2.71 [2.38, 3.06]	−0.5 [−0.85, −0.15]*	−0.35 [−0.55, −0.16]**	−0.5(−0.5)
9 months	2.48 [2.23, 2.74]	2.47 [2.13, 2.82]	−0.16 [−0.53, 0.21]	−0.26 [−0.48, −0.03]*	
Readiness for change					
Baseline	3.56 [3.38, 3,75]	3.49 [3.23, 3.76]			
1 month	3.61 [3.42, 3.8]	3.29 [3.01, 3.56]	0.26 [−0.13, 0.64]	0.05 [−0.17, 0.26]	
9 months	3.57 [3.35, 3.79]	3.21 [2.92, 3.5]	0.29 [−0.12, 0.71]	0.01 [−0.24, 0.25]	
Bystander intent					
Baseline	3.7 [3.53, 3.87]	3.58 [3.33, 3.83]			
1 month	3.9 [3.73, 4.07]	3.4 [3.15, 3.65]	0.37 [0.09, 0.65]*	0.2 [0.04, 0.35]*	1(0.99)
9 months	3.91 [3.72, 4.1]	3.47 [3.21, 3.73]	0.31 [0.01, 0.61]*	0.21 [0.03, 0.38]*	0.94(0.93)
Bystander efficacy					
Baseline	80.8 [75.67, 85.93]	81.75 [74.22, 89.28]			
1 month	87.07 [81.94, 92.2]	73.73 [66.2, 81.25]	14.29 [6.37, 22.22]***	6.27 [1.85, 10.69]*	0.9 (0.89)
9 months	87.36 [81.7, 93.01]	73.93 [66.27, 81.59]	14.38 [5.98, 22.78]***	6.56 [1.55, 11.56]*	0.98 (0.97)
Bystander behavior					
Baseline	−2.52 [−4.3, −0.62]	−3.25 [−5.96, −0.54]			
1 month	−0.21 [−2.06, 1.65]	−3.67 [−6.47, −0.88]	2.69 [−1.86, 7.23]	2.26 [−0.27, 4.8]	
9 months	0.69 [−1.56, 2.94]	−2.76 [−05.64, 0.13]	2.7 [−02.12, 7.46]	3.16 [0.31, 6.02]*	
Perceptions of peer helping					
Baseline	3.71 [3.52, 3.91]	3.84 [3.55, 4.12]			
1 month	4.01 [3.82, 4.21]	3.74 [3.42, 4.05]	0.4 [−0.04, 0.84]	0.3 [0.06, 0.54]*	
9 months	3.99 [3.75, 4.23]	3.87 [3.57, 4.18]	0.24 [−0.22, 0.7]	0.28 [−0.001, 0.56]	
Perceptions of peer myths					
Baseline	29.76 [24.79, 34.73]	32.5 [25.08, 39.94]			
1 month	22.05 [17.08, 27.02]	27.89 [20.22, 35.56]	−3.09 [−14.56, 8.38]	−7.71 [−13.93, −1.49]*	
9 months	28.98 [22.9, 35.06]	30.52 [22.93, 35.03]	1.22 [−10.74, 13.17]	−0.78 [−7.95, 6.39]	
The law					
Knowledge of law DV					
Baseline	2.68 [2.4, 2.95]	2.25 [1.85, 2.65]			
1 month	3.43 [3.15, 3.7]	2.62 [2.22, 3.03]	0.37 [−0.17, 0.92]	0.75 [0.44, 1.06]***	
9 months	3.16 [2.84, 3.48]	2.45 [2.04, 2.87]	0.28 [−0.3, 0.86]	0.48 [0.14, 0.83]*	
Knowledge of law SV					
Baseline	2.79 [2.5, 3.09]	2.44 [2.002, 2.87]			
1 month	3.59 [3.29, 3.89]	2.85 [2.4, 3.3]	0.38 [−0.23, 1]	0.79 [0.45, 1.14]***	
9 months	3.39 [3.04, 3.74]	2.79 [2.33, 3.25]	0.24 [−0.41, 0.89]	0.6 [0.21, 0.99]*	

*Note*. CI = confidence interval; IRMA = Illinois Rape Myth Acceptance Short Form; DVMAS = Domestic Violence Myth Acceptance.

a Estimates based on linear mixed models.

b T0 = baseline, T1 = post-test, T2 = follow-up.

c Standardized calculation based on model-adjusted means in both groups.

* *p* < .05. **p < .01. ***p < .001.

In terms of frequency of bystander opportunity, descriptive analysis showed that intervention group participants with opportunity to intervene reported engaging in more bystander behaviors at post-test and follow-up compared with the control group participants who engaged in fewer behaviors over time (Table S4, Supplemental Appendix).

##### Within-Group

Analysis of the intervention effects within the intervention group revealed significant improvements with medium to large effect sizes on IRMA scores at post-test, *d*(*g*) = −0.68(−0.67) and at follow-up, *d*(*g*) =−0.88(−0.88), and DVMAS scores at post-test, *d*(*g*) = −0.53(−0.53), and at follow-up, *d*(*g*) = −0.4(−0.4). Participants significantly improved on their Bystander Intent at post-test, *d*(*g*) = 0.39(0.39) and at follow-up, *d*(*g*) = 0.43(0.43), and Bystander Efficacy at both time points, *d*(*g*) = 0.42(0.41) and *d*(*g*) = 0.45(0.45), respectively. Law knowledge related to DV and SV was also significantly greater with large effect sizes at post-test, *d*(*g*) = 0.93(0.92) and *d*(*g*) = 0.91(0.9) respectively, and at follow-up, *d*(*g*) = 0.62(0.61) for DV, and *d*(*g*) = 0.69(0.69) for SV. After one month, participants significantly improved on their Perceptions of Peer Helping, *d*(*g*) = 0.52(0.51), and Peer Myth Acceptance, *d*(*g*) = −0.53(−0.52), but not at follow-up. No significant changes were observed for other measures.

#### Secondary Outcomes

##### Backlash

We identified backlash in 9.38% of the intervention group for bystander efficacy post-test (*n* = 3) and 4.35% at follow-up (*n* = 1). These changes in the undesired direction are outweighed by the proportion of participants whose scores improved by at least 1 *SD* post-test (75%) and at follow-up (86.96%). We also observed backlash in 4.55% of the intervention group in perceptions of peer helping at follow-up (*n* = 1), outweighed by a proportion of participants (4.55%) whose scores improved by at least one SD on this measure, while there were no differences in the control group (χ^2^ = 1.25, *p* = .53).

## Discussion

The purpose of this study was to examine the extent to which the exposure to the Football Onside program had effects on participants’ knowledge, attitudes, and confidence about sexual and domestic violence, as well as their bystander behaviors. We also examined if the program learning objectives were met, and observed fidelity to the program. To our knowledge, this was the first UK-based study exploring the effects of a bystander intervention for the prevention of sexual and domestic violence in a professional sports setting. The results suggest mixed but promising changes and provide preliminary support for both the efficacy of the Football Onside program in UK population settings and the premise that professional sports may be an appropriate and positive platform for VAWG prevention. The results also provide further evidence for the translatability of bystander interventions from the U.S. context (Fenton & Mott, 2017). These findings should be investigated in larger cluster randomized controlled trials.

The consistently high ratings for self-reported learning outcomes and the observed high engagement with the program suggests that content and mode of delivery were appropriate for the target audience. Further, as our participants were almost exclusively men, a traditionally hard to engage, but critical, group (Casey et al., 2018; Flood, 2011) is important for educators exploring bystander programs in real-world professional settings and adds to the evidence that some men will positively receive prevention training (Rich et al., 2010). This finding attests to both the potential of professional sports settings as critical spaces for prevention (Katz, 2018) and the importance of the bystander approach in positioning men as “social justice allies” (Fabiano et al., 2003). The need to replace facilitators highlights the importance of careful selection and thorough training of even professional facilitators, which will be essential for the delivery, engagement, and sustainability of future programs (Anderson & Whiston, 2005; Fenton & Mott, 2017; Nation et al., 2003).

There were several challenges to program and study implementation. Staff availability during the busy sports season was limited, presenting difficulty in releasing staff to take part in the evaluation and subsequent allocation of sufficient time for research activities. Researchers should be mindful of high staff turnover rates at Community Trusts (Bostock et al., 2021)—a factor beyond our control that was the main reason for study attrition. Securing buy-in to participation in the program in Trust 1, however, was not a challenge itself because it was presented by the CEO as part of staff responsibilities. This active involvement and positive role-modeling by the CEO, and stakeholders, further facilitated participation in the program and study. This aligns with similar findings by Fields et al. (2022), where support from athletic directors and athletes enhanced the sense of community and increased participation in CBIM.

A further facilitator to participant engagement was the cohort of people at which Football Onside’ was aimed. Those working at the Community Trust were generally very community-minded because this is at the core of their activities. Delivering the program to a cohesive peer group with similar job responsibilities and perceived prosocial attitudes underscored the importance of giving space for participants and “the opportunity to gather with like-minded men” to engage with VAWG (McMahon & Dick, 2011).

While the overall effects of the intervention were mixed, significant changes were observed for the measures that in particular correlate with the fundamental theoretical design of the Football Onside program (long-term improvement for rape myth acceptance, bystander intent and confidence, and short-term improvement for domestic violence myth acceptance), with the exception of the actual helping behavior stage. These measures map well onto the processes of Latané and Darley’s (1970) situational model as applied to VAWG, and previous studies (Jouriles et al., 2018; Moynihan et al., 2011a).

The maintained long-term improvement in rape myth acceptance speaks to the importance of meeting participants where they are at (Fenton & Jones, 2017): indeed, baseline RMA data were used to inform program development (Fenton et al., forthcoming). We do not know exactly why the significant improvements in DVMA were not maintained after 9 months but suspect it may be due to the fact that the program focused more explicitly on rape myths, in part because so much more is known from rape myth research. To our knowledge, domestic abuse myths have not yet been measured in evaluations of bystander programs with athletes; hence, further research should investigate change processes for this outcome.

Evidence that Football Onside may increase Bystander Intent and Efficacy is especially promising since these measures are important correlates of prosocial bystander behavior (Banyard, 2008). Moynihan et al. (2011a) observed similar improvements in a student athlete sample, but only in a 2-month follow-up evaluation. Our follow-up period of 9 months, however, is comparatively longer than the vast majority of evaluations (Kovalenko et al., 2022; Mujal et al., 2021), indicating potential for long-lasting change.

The final step in Latané and Darley’s (1970) theoretical model is progression to actual bystander behavior. There were no significant differences in behavior between groups, although trends were in the desired direction. Descriptive analysis showed an increase from baseline to follow-up in bystander actions (when in the situation) in the intervention group, when participants have had more substantial time and opportunity to enact interventions. Many other studies have reported nonsignificant effects for bystander behavior (e.g., Jouriles et al., 2018; Moynihan et al., 2011a), including a male-targeted bystander program (Gidycz et al., 2011) and a student athlete sample (Moynihan et al., 2018). The trends within our results suggest that our study may simply have not been sufficiently powered to show significant differences. Behavior is notoriously difficult to change and there are challenges in evaluating bystander behavior. Firstly, at no time point did more than half of either group report being in the described situation, thus limiting the possibility to observe the intervention effects and possibly indicating an under-capture of bystander behaviors. Secondly, despite several attempts to measure opportunity (Cares et al., 2015; McMahon, 2015), this construct is still not fully captured with the existing measures and calculations. Descriptive analysis of the “yes,” “no,” and “wasn’t in the situation” options allows the assessment of frequency of opportunity and behaviors. However, summing the scores for inferential statistical analysis does not allow for meaningful conclusions about individual behavioral changes to be drawn, especially as some events are rare. Yet if analyzed separately, multiple comparisons of individual behaviors would introduce an increased chance of Type I error. Given the complexity of real-life opportunities to intervene and the diverse range of prosocial behaviors that might be performed, it is unlikely that quantitative methodology can ever prescriptively capture them. Inductive thematic analysis of additional open-ended survey questions (Braun & Clarke, 2006) or interviewing could be a possible solution. Nonetheless, real-life impact at a societal level can be cumulatively achieved by small individual effects (Jouriles et al., 2018).

That participants’ Readiness did not significantly improve might be explained by the fact that their prior safeguarding training and job roles meant that they were already ready for change. However, given that our participants also had desirable baseline RMA scores, which did improve significantly after 9 months, desirability at baseline does not necessarily equate with no room for improvement. Further, as other studies have found an association between RMA and denial and taking responsibility (Banyard et al., 2014; Fenton & Mott, 2017), we suggest that further examination is required. We speculate that items on the Readiness scale, such as “I think I should learn more,” might not fit the construct at post-intervention when participants have indeed just learned more; they may logically think that they should not learn more because the depth of the intervention means they have learned enough. Further, the presentation to participants of their own misperceptions of norms as a pedagogic device in the intervention might actually serve to increase denial. This is because participants now understand that others are more prosocial and hold fewer problematic views than they originally thought, and thus logically that it is less “of a problem in their organization.” We suggest that the items and construct underpinning Readiness should be explored in more detail in further research.

The lack of significant difference between groups on Perceptions of Peer Helping and Perceptions of Peer Myth Acceptance is inconsistent with previous research, which has shown that correcting negative perceptions of peer norms is associated with personal willingness to intervene (Brown & Messman-Moore, 2010; Fabiano et al., 2003). Our finding may well attest to the difficulties in measuring the concept of peers generally. For example, individuals have multiple social identities and identify with multiple social groups, within which different norms may operate (Turner & Reynolds, 2010). The level of social identification with each group may influence their understanding and perception of “peers,” and peer norms. We used “friends, family and colleagues,” which was unvalidated, and acknowledge that this is a wide and possibly confusing comparator group requiring further development and refinement as a measure. However, the other purpose of this measure was to be able to present participants with their own misperceptions of norms as part of the intervention in accordance with social norms theory (Orchowski & Berkowitz, 2022). We note from the observation (and years of facilitator experience) that as a pedagogical device, this strategy results in high interest and engagement among participants. Equally, it may be simply that our sample was underpowered.

We found no significant differences in perceived law knowledge between groups. To our knowledge, no studies have extensively measured law-related knowledge, although it is often part of program curricula (Fenton et al., 2016). Thus, its effect on change processes is unknown. A validated law scale would be a valuable addition to the literature expanding understandings as to how bystander programs work.

Additional changes that were observed only on the within-group level included significant increases in Perceived Law Knowledge related to DV and SV in the intervention group at both time points, and bystander behavior at follow-up. Very few participants evidenced a backlash effect. Worsened scores on the measures of bystander efficacy and perceptions of peer helping were outweighed by a higher proportion of scores improved as a result of participation in Football Onside.

### Limitations

Our study has several limitations that should be addressed in future research. Our sample could inhibit generalizability of findings to a broader population for several reasons. First, it was a UK-specific study, and findings may not apply to international contexts where the structure and functions of sports and charity organizations are different from the UK FCCT’s. Second, the sample size was small and was also underpowered to reliably detect meaningful differences between groups, and we additionally lost a large number of participants to follow-up due to unanticipated staff turnover. This also means we were unable to look at the interactions of gender and age on the outcomes of interest to produce meaningful results. However, a large cluster randomized trial could address this limitation, as well as treatment assignment bias. If participants are randomly assigned and are blind to condition, this would reduce the chance of social desirability and researcher bias. Third, multiple comparisons in a small sample introduce a higher chance of Type I error. We adopted a conservative approach with Kenward–Roger approximation to minimize this issue; however, the results should be interpreted with caution. Fourth, our study sample was relatively homogenous with respect to racial/ethnic identity and sexual orientation. However, previous research has shown that White heterosexual men are the bearers of hegemonic masculinity (Carrigan et al., 1985; Donaldson, 1993; Jewkes et al., 2015); hence, we argue that in the current study, it is a strength rather than a limitation. Fifth, reliance on retrospective self-reports can result in underestimates or overestimates in these measures and has been noted by other researchers (Sharot et al., 2007). Participants might not recall certain situations, or the memory could be distorted by emotions. They might also misunderstand and consequently underestimate their bystander involvement. Sixth, we calculated standardized effect sizes based on model-adjusted means between two groups. To date, there have been no guidelines on the calculation of effect sizes from mixed-effects models. More research is needed to advance reporting. Other limitations were introduced by providing a long questionnaire with modified scales. One of the reasons for dropouts at follow-up could be related to the length of the questionnaire. Although our study found acceptable internal consistency for each scale in our sample, the use of replicated items and modified language should be investigated further. Despite the limitations, however, we find it encouraging that the participants reported having a chance to intervene and giving examples of doing so since the beginning of the program.

## Conclusions

The current feasibility study evaluated a bystander violence prevention program at FCCTs in South West England. The Football Onside program appears to have a promising impact on Community Trust members’ attitudes and confidence to intervene. These findings are among the first to promote bystander intervention in professional football club settings. The two Community Trusts represented an ideal population for the study due to the outreach and impact on young people and communities. Trust coaches as leaders could be positive role models promoting new prosocial norms among their players. Further, this study expands the research base for bystander programs by recruiting a predominantly adult male sample with a mean age of 31. Further research should explore intervention effects through a cluster randomized controlled trial in professional athlete teams.

## Supplemental Material

sj-docx-1-jiv-10.1177_08862605241239452 – Supplemental material for Bystander Intervention in Football and Sports. A Quasi-Experimental Feasibility Study of a Bystander Violence Prevention Program in the United Kingdom Supplemental material, sj-docx-1-jiv-10.1177_08862605241239452 for Bystander Intervention in Football and Sports. A Quasi-Experimental Feasibility Study of a Bystander Violence Prevention Program in the United Kingdom by Anastasiia G. Kovalenko and Rachel A. Fenton in Journal of Interpersonal Violence

## Notes

1. Community Trusts are not for profit organizations that support health and wellbeing within their local community through involvement in sports and health projects. CT’s are usually recognized among other supporters and nonsupporters in the United Kingdom due to their extensive outreach (Martin et al., 2016; Pringle et al., 2021).
