# Supplementary material for: Bystander Intervention in Football and Sports. A Quasi-Experimental Feasibility Study of a Bystander Violence Prevention Program in the United Kingdom
Source: J Interpers Violence. 2024 Mar 26;39(21-22):4598–625. doi: 10.1177/08862605241239452 (PMC11465711; doi:10.1177/08862605241239452)
Supplement: sj-docx-1-jiv-10.1177_08862605241239452 – Supplemental material for Bystander Intervention in Football and Sports. A Quasi-Experimental Feasibility Study of a Bystander Violence Prevention Program in the United Kingdom [file sj-docx-1-jiv-10.1177_08862605241239452.docx]

Supplementary material

*Table S1. Program content*

| Content and format | Situational Model |
| --- | --- |
| Three 2-hour sessions  1x week |  |
| *Session One (2 hours):*   - Introduction to bystander and social norms theories - Performative masculinities, root causes of VAWG   *Format:* presentation, videos, individual and group tasks, discussion | Increased knowledge and ability to notice an event  Increased motivation  Increased sense of responsibility  Increased self-efficacy  Increased willingness and intent to act |
| *Session Two (2 hours):*   - Performative masculinities - Rape and DV myths - Consent - Impact and empathy towards victims - In-depth understanding of law relating to sexual offences and child sexual offences, warning signs of DV, grooming, consent (and production of a take-away booklet)   *Format:* presentation, videos, individual and group tasks, discussion | Increased knowledge and ability to notice an event and identify as problematic  Increased motivation  Increased sense of responsibility  Increased self-efficacy  Increased willingness and intent to act |
| *Session Three (2 hours):*   - Own misperceptions of norms - Intervention strategies - Practice scenarios   *Format:* presentation, videos, individual and group tasks, discussion, behavior rehearsal (role play) | Increased self-efficacy  Possesses the necessary skills to act  Increased knowledge and ability to notice an event  Increased motivation  Increased sense of responsibility  Increased willingness and intent to act |

*Table S2. Baseline characteristics of the full sample.*

| **Characteristic** | | **Trust 1**  **Intervention** | **Trust 2**  **Control** |
| --- | --- | --- | --- |
| N=50 | | 34 | 16 |
| Age, M | | 30.03(10.626) | 34.53(11.363) |
|  |  | %(n) | %(n) |
| **Gender**  Male  Female  Other | | 88.2% (30)  8.8% (3)  3% | 75% (12)  25% (4) |
| **Orientation**  Heterosexual  Gay woman  Other  Prefer not to say | | 85.3% (29)  5.9% (2)  2.9% (1)  5.9% (2) | 100% (16) |
| **Ethnicity**  White British  Mixed White and Black Caribbean  Other White background | | 97.1% (33)  2.9% (1) | 93.8% (15)    6.2% (1) |
| **Relationship status**  In a relationship  Not in a relationship | | 55.9% (19)  44.1% (15) | 75% (12)  25% (4) |
| **Do you know someone who has been affected?**  Yes  No  Unsure | | 41.2% (14)  50% (17)  8.8% (3) | 43.8% (7)  43.8% (7)  12.4% (2) |
| **Have you participated in a DV or SV program before?**  Yes  No  Unsure | | 5.88% (2)  91.18% (31)  2.94% (1) | 18.75% (3)  81.25% (13) |
| **Have you participated in a DV or SV campaign before?**  Yes  No  Unsure | | 2.94% (1)  97.06% (33) | 12.5% (2)  81.25% (13)  6.25% (1) |

*Table S3. Mean participant feedback score against learning objectives*

| The programme met its objectives in assisting me to: | M | SD |
| --- | --- | --- |
| Improve knowledge about sexual violence, harassment, and domestic abuse | 4.12 | .977 |
| Understand that violence against women is a serious problem in society | 4.38 | .779 |
| Understand that criminal offences and other law relating to SV/harassment and DV | 4.18 | .757 |
| Understand that ‘banter’ is harmful | 4.29 | .76 |
| Know of where to seek help and support in cases of domestic abuse | 4.35 | .691 |
| Know of where to seek help and support in cases of sexual violence | 4.41 | .743 |
| Understand the stages of bystander interventions from noticing to acting | 4.64 | .548 |
| Understand that individuals can often be mistaken about others’ beliefs and values | 4.42 | .614 |
| Be familiar with intervention strategies | 4.29 | .76 |
| Be confident to use intervention strategies in their everyday life | 4.24 | .741 |
| Increase the likelihood they would use intervention strategies in their everyday lives | 4.15 | .857 |
| **Overall mean** | **4.32** | **.575** |

*Table S4. Percentage of situations when participants had an opportunity to intervene vs the percentage of situations when participants intervened.*

| **Group** | **Had Opportunity,**  **% situations** | | | **Intervened,**  **% situations** | | |
| --- | --- | --- | --- | --- | --- | --- |
|  | Baseline | 1 month | 9 months | Baseline | 1 month | 9 months |
| Intervention | 35% | 25.8% | 20.1% | 26% | 48% | 57% |
| Control | 49% | 37.5% | 39% | 31% | 22.5% | 28.7% |

# 
